# Thermodynamic Parameters of Crosslinked Elastomers (BR, SBR and NBR) and Their Blends

**DOI:** 10.3390/polym16030351

**Published:** 2024-01-28

**Authors:** César Leyva-Porras, Iván A. Estrada-Moreno, Claudia I. Piñón-Balderrama, Sergio G. Flores-Gallardo, Alfredo Márquez-Lucero

**Affiliations:** Advanced Materials Research Center (CIMAV), Complejo Industrial Chihuahua, Miguel de Cervantes No. 120, Chihuahua 31136, Mexico; claudia.pinon@cimav.edu.mx (C.I.P.-B.); sergio.flores@cimav.edu.mx (S.G.F.-G.); alfredo.marquez@cimav.edu.mx (A.M.-L.)

**Keywords:** thermodynamic parameters, elastomer blends, Flory–Huggins interaction parameter, swelling of crosslinked rubbers

## Abstract

Herein, a methodology is employed based on the Flory–Rehner equation for estimating the Flory–Huggins interaction parameter (χ_12_*) of crosslinked elastomer blends. For this purpose, binary elastomer blends containing polybutadiene rubber (BR), styrene–butadiene rubber (SBR) and nitrile–butadiene rubber (NBR), were prepared in a mixing chamber at a temperature below the activation of the crosslinking agent. Swelling tests with benzene were employed to determine the crosslinked fraction, finding that after 20 min of thermal annealing, the BR and NBR were almost completely crosslinked, while the SBR only reached 60%. Additionally, the BR-SBR blend increased by 2–3 times its volume than its pure components; this could be explained based on the crosslink density. From the mechanical tests, a negative deviation from the rule of mixtures was observed, which suggested that the crosslinking was preferably carried out in the phases and not at the interface. Furthermore, tensile tests and swelling fraction (ϕ_sw_) results were employed to determine the average molecular weight between two crosslinking points (M_c_), and subsequently χ_12_*. Calculated χ_12_* values were slightly higher than those reported in the literature. The calculated thermodynamic parameters for the blends showed positive ΔG_mix_ values and endothermic behavior, suggesting their immiscible nature.

## 1. Introduction

In recent years, elastomers have been used as an integral part in the development of chemical sensors and actuators, such as artificial noses, distributed sensors, and superabsorbent materials [1,2,3]. In this type of application, the ability of these elastomers to absorb low-polarity substances is of great importance and one of their great advantages. Currently, there is little literature about methods to increase the absorption capacity of these substances by elastomers. However, there is a large amount of literature on super-absorbent hydrogels for aqueous solutions [4,5]. There are many ways to obtain new polymers that present certain superior physicochemical properties, but commonly, these processes involve complicated and expensive polymerization reactions. On the other hand, sometimes it is only necessary to mix the polymer with some other material that may impart new properties [6]. Elastomer blends are important since they present better performance in their final properties, which are not achievable by the individual elastomers [7]. For example, among the well-known properties of hydrogenated acrylonitrile-butadiene rubber (HNBR) are chemical and thermal degradation, while the use of chloroprene rubber (CR) provides the flame resistance property. The blending of these rubbers when crosslinked with silver oxide (Ag_2_O) improved the homogeneity and miscibility, resulting in a material with significant resistance to thermo-oxidative factors [8]. Within the field of polymer blends, several works have reported the behavior of miscible and immiscible blends, variations in polarity and unsaturation, differences in molecular weights and branching of polymers [9,10].

Rubbers such as polybutadiene (BR), poly(butadiene-*co*-styrene) (SBR) and poly(butadiene-*co*-acrylonitrile) (NBR) are of great importance in the production of tires, engineering resins, adhesives, seals, hoses and gaskets, and in materials that are oil resistant. Globally, its demand requires a consumption of approximately 58% of the total production of butadiene, which is used as monomer for their production [11,12,13]. The study of mixing any of these three elastomers with other polymers has been studied extensively [14,15,16,17,18,19,20,21]. However, works reporting PB-NBR and SBR-NBR blends are scarce, particularly PB-SBR. For example, blends of SBR and NBR have been used to promote the uptake of chlorinated hydrocarbons, where the polarity of the hydrocarbon and the SBR fraction affect the degree of absorption [22]. Laser scanning confocal microscopy (LSCM) was used to demonstrate phase separation in PB-SBR blends. It was found that the SBR domains presented a fine structure of the order of sub-micrometers, while the continuous phase of PB presented domains separated by 10 μm [23]. The incorporation of *trans*-polyoctylene rubber (TOR) improved the mechanical properties and crosslinking density of SBR-recycled NBR (rNBR) blends. This is because the compatibilizer agent improves the adhesion between the SBR and rNBR phases [24]. In this sense, the addition of mesoporous silica particles as a compatibilizer agent improves the mechanical behavior of SBR-NBR blends, since the silica surface promotes the dispersion of the NBR phase [25]. Because of the high surface area and the presence of polar groups on the surface of the nanofillers, the physical interactions and interfacial adhesion between blends of BR-SBR-carbon black containing isocyanate modified graphite nanoplatelets (i-MG) were enhanced, showing an increase in mechanical and thermal properties, hardness and abrasion resistance [26]. In the case of carboxylated NBR and SBR blends reinforced with graphene oxide (GO), the increase in the GO content causes an increase in the crosslinking density and consequently a decrease in the swelling ratio, which makes the XNBR-SBR blend more resistant to solvents [27]. In 70/30 SBR/BR blends with silica as filler, the increase in the content of free fatty acids (FFA) in palm oil (PO) increased the crosslinking density, which was reflected in the increase in mechanical properties. such as modulus, tensile strength and abrasion resistance [28]. In SBR-NBR blends where the SBR was modified with dichlorocarbene, the degree of swelling in the presence of *n*-heptane decreased with the NBR content, from 75.3 to 59.9 and 24.3%, for the 70/30, 50/50 and 30/70 of SBR/NBR blends, respectively. This was caused by the polar character of NBR, which restricts swelling [29].

One of the most important reasons for the use of compatible blends is the ease with which they are prepared, since only involve the use of a mixing instrument and raw materials. For example, sensors have been developed to detect hydrocarbon leaks, the principle of which is based on the swelling of the polymer upon contact with the hydrocarbon [30]. This swelling must not only be rapid, but must also have certain properties such as mechanical resistance. Because the glass transition temperature of elastomers is below room temperature, they are flexible, while at temperatures above 80 °C they are viscoplastically deformable. One way to impart rigidity to the structure is through chemical reactions in the solid state, known as a crosslinking reaction, which results in a three-dimensional network that prevents the movement of the polymer chains. The crosslinking reaction is largely determined by the type of crosslinking agent, process, temperature and time [31]. The number of crosslinks formed is known as the crosslink density and has a large influence on the final mechanical properties of the elastomer. Blends are commonly used to improve the processability of rubbers. This improvement may consist of either lowering the viscosity or producing a material less prone to fracture during melt flow. Side effects such as swelling of an extrudate after leaving a forming die can also be affected by mixing.

The importance of knowing the thermodynamic parameters of elastomer blends lies in the possibility of predicting their miscibility. Phase homogeneity in rubber mixtures is promoted when both individual components share properties such as viscosity, solubility parameters and polar groups [32]. The equilibrium miscibility criterion for a polymer blend is given by the Gibbs free energy of mixing (ΔG_mix_), which must be negative; while for the stability of the mixture, its second derivative with respect to the concentration must be positive [33]. For a polymer–solvent system, the thermodynamic properties are calculated from the Flory–Huggins model, using solubility values to estimate the interaction parameter (χ). However, although this equation is widely used due to its simplicity, it does not allow the interaction parameter for polymer blends exposed to a solvent to be calculated. Therefore, it is necessary to find a method that allows the average interaction parameter for polymer blends subjected to the absorption of solvents to be estimated.

Thus, in the present work, a phenomenological methodology based on the Flory–Rehner equation is presented to determine the interaction parameter of crosslinked binary elastomer blends and the calculation of the thermodynamic parameters. This approach is based on swelling experiments with an organic solvent and the measurement of the mechanical properties from specimens of the blends. The contribution of this work relies on the calculation of the Flory–Huggins interaction parameter and the thermodynamic parameters of elastomer blends of immiscible nature.

## 2. Physicochemical Models

The Flory–Huggins model is based on the division of ΔG_mix_ into enthalpic and entropic terms, and the evaluation of these two terms separately according to Equation (1):

ΔG_mix_ = ΔH_mix_ − TΔS_mix_ (1)

The entropy of mixing (ΔS_mix_) is evaluated from the possible number of arrangements of the molecules in the network. The mixing enthalpy (ΔH_mix_) is calculated as the change in the interaction energies between the molecular surfaces during the mixing process. These terms are defined as follows:

ΔS_mix_ = −R(*n*_1_lnϕ_1_ + *n*_2_lnϕ_2_) (2)

ΔH_mix_ = RT *n*_1_ϕ_2_χ (3)

where the volume fractions ϕ_1_ and ϕ_2_ represent the total number of lattice points occupied by solvent molecules and polymer segments, respectively, *n*_1_ is the number of moles of solvent, *n*_2_ is the number of moles of polymer, and χ is the Flory interaction parameter [34].

The polymer–solvent binary system is based on the network model, where the effect of the difference in size between the polymer and solvent molecules on the mixing entropy is pointed out. The quantitative calculation of the mixing enthalpy allows the introduction of a dimensionless value called the Flory–Huggins interaction parameter, for the thermodynamic description of polymer solutions. This parameter considers the specific interactions between polymer segments and solvent molecules within a particular system. Then, after substituting the entropy and the enthalpy of mixing in Equation (1), the well-known Flory–Huggins relationship is obtained (Equation (4)) [35]:

ΔG_mix_ = RT (*n*_1_lnϕ_1_ + *n*_2_lnϕ_2_ + *n*_1_ϕ_2_χ) (4)

One of the simplest ways to calculate the interaction parameter is employing the solubility parameters of the polymer and the solvent (Equation (5)), where V_1_ is the molar volume of the solvent, and δ_1_ and δ_2_ are the solubility parameters of the polymer and the solvent, respectively [36]:

Χ = 0.34 + V_1_/RT(δ_1_ − δ_2_)^2^ (5)

According to this equation, the concept of the solubility parameter implies that the interaction parameter must always be positive, while the lowest possible value is 0.34, applicable to mixtures of components with exactly the same value of the solubility parameters. However, this equation, although it is a widely used approximation due to its simplicity, does not allow the interaction parameter for polymer blends exposed to a solvent to be calculated. For describing the elasticity phenomenon of crosslinked rubbers subjected to swelling due to the absorption of a solvent, the Flory–Rehner Equation (6) is used [36]:

ln(1 − ϕ_sw_) + ϕ_sw_ + χ _12_ϕ_sw_^2^ + (ρV_1_/M_c_)(ϕ_sw_^1/3^ − ϕ_sw_/2) = 0 (6)

where ϕ_sw_ is the inverse of the swelling ratio of V_0_ and V, the volumes of the dry and swollen gel, respectively. ρ is the density of the polymer, V_1_ is the molar volume of the solvent, χ_12_ is the Flory–Huggins interaction parameter of the blend and M_c_ is the average chain size between two crosslinks.

Additionally, from the stress–strain curves, it is possible to determine the effect of the nature and distribution of the crosslinks in the rubber, since changes in the module, elongation and stress are influenced by alterations in the structure of the polymeric chains [37]. Now, the shear modulus (G) of a rubber is a direct measure of the number of elemental chains in a unit of volume (N_v_), as

G = kTN_v_ (7)

with k being the constant of Boltzmann and T the temperature. Frequently, the number of chains is expressed through the average molecular weight of the chain between two crosslinks (M_c_), rubber density (ρ) and the universal gas constant (R). The result is:

G = RTρ/M_c_ (8)

Then, by determining the Young’s modulus (E) and taking into account that the relationship of the shear modulus with the Young’s modulus is G = E/3, the molecular weight between two crosslinking units can be estimated from tensile tests [35] as

M_c_ = 3RTρ/E (9)

Then, from the swelling experiments, the swelling ratio can be determined, while from the mechanical tests, the average molecular weight between two crosslinks is determined. Knowing both parameters, the Flory–Huggins interaction parameter of the blend is calculated, solving the Flory–Rehner equation.

## 3. Materials and Methods

### 3.1. Materials

Polybutadiene rubber (BR) (Solprene 200, Dynasol, Altamira, Mexico), poly(butadiene-*co*-styrene) (SBR) (Emulprene 1778, Industrias Negromex, Altamira, Mexico) and poly(butadiene-*co*-acrylonitrile) (NBR) (Paracril BJLT-M50, Quimipol, Mexico city, Mexico) were used as raw materials for preparing the rubber blends. Dicumyl Peroxide (DCP) (99.02%, Kaucho Quimico, Mexico city, Mexico) was employed as the crosslinking agent. Benzene (99.9%, Merck, Naucalpan de Juarez, Mexico) was employed as the solvent for the Soxhlet extractions and swelling tests.

### 3.2. Rubber Blends Preparation

The mixing of the rubbers with the crosslinking agent was carried out in an internal mixing chamber (Brabender, South Hackensack, NJ, USA). A chamber type VI was used with a gear ratio of 3:2, and a volume capacity of 60 cm^3^ when using Cam-type blades. Heating and cooling were carried out through an electrical resistance and circulating air, respectively. Mixing was carried out at a temperature of 90 °C, and 40 RPM, for 15 min. The mass of the rubber (60 g) was added and mixed for 2 min. At this time, 1 phr (parts per 100 of resin) of the crosslinking agent was added into the chamber. At the end of the mixing time, the sample was removed from the chamber and identified according to Table 1:

### 3.3. Crosslinking

The crosslinking of the rubber samples with DCP was carried out in a forced air heating oven (LindBerg Blue, Thermo Fisher Scientific Inc., Mexico city, Mexico), with a precision temperature controller of ±0.1 °C. The crosslinking was carried out at 175 °C, and different times of 2, 4, 6, 10 and 20 min. This crosslinking temperature was selected to ensure at least a 50% crosslinking efficiency. These experiments were carried out by duplicate.

### 3.4. Crosslinked Fraction in the Blend and Swelling Tests

Once the samples were crosslinked, the gel fraction or the crosslinked fraction of the blends was calculated. For this, a solid–liquid Soxhlet extraction was carried out, with benzene as solvent. The extraction process was repeated for 24 h, to ensure that all the non-crosslinked fraction was dissolved. The sample was then dried at 90 °C for 24 h, to evaporate the solvent. Subsequently, dried sample was weighed and the crosslinked fraction (Θ) was determined according to Equation (10):

Θ = [(W_4_ − W_1_)/W_2_] × 100 (10)

where W_1_ is the initial weight of the extraction capsule, W_2_ is the weight of the rubber sample and W_4_ is the dried weight of the remaining rubber sample in the capsule after extraction process.

Once the Soxhlet extraction was completed and the solvent was cooled to room temperature, the excess of solvent was removed using absorbent paper, then the capsule containing the swollen elastomer was weighed. The weight was recorded as W_3_. The swelling ratio or the volume of solvent absorbed per unit volume of crosslinked polymer (1/Φ_sw_ = V/V_0_) was determined according to Equation (11):

(11) $$1/\Phi_{\mathrm{sw}}=V/V_{0}=1+\frac{W_{3}-W_{4}}{W_{4}}\left(\frac{\rho_{2}}{\rho_{1}}\right)$$

where W_3_ is the swollen mass of the rubber and solvent, ρ_1_ and ρ_2_ are the densities of the solvent (benzene = 880 kg/m^3^) and the elastomer sample (values reported in Table S1), respectively [38]. The value of 1/Φ_sw_ indicates the times the initial volume of the rubber increases when it is immersed in the solvent. These experiments were carried out in duplicate.

### 3.5. Tensile Tests

Tensile tests were carried out on an Instron Universal Machine, model 4468 (Norwood, MA, USA) with a 500 kg load cell. The specimens and the tension test were carried out in accordance with the ASTM D-638 standard [39], with dimensions of the specimens of 12 cm × 2 cm × 0.3 cm, deformation rate of 500 mm/min and temperature of 25 °C. Young’s modulus was determined from the ratio of stress and deformation (E = σ/ε).

### 3.6. Density of Rubber Blends

To determine the density of the samples, an Ultrapycnometer 1000 (Quantachrome, Boynton Beach, FL, USA) was used. The instrument determines the density of a solid from the displaced volume of Helium gas.

## 4. Results and Discussion

### 4.1. Crosslinked Fraction and Swelling Ratio

Table S1 shows the values of the masses of the elastomers and their blends during the Soxhlet extraction process, the calculated crosslinked fraction and density values. Clearly, there is no significant effect of the initiator on the density of the samples, because the volume of the sample remains constant during crosslinking, and only some chemical bonds change position when the hydrogen extraction is carried out by the radical peroxide. Furthermore, NBR showed the highest density value (980 Kg/m^3^), while SBR and BR showed the lower values of 930 and 890 (Kg/m^3^), respectively. On the other hand, when calculating the density for the different blends, intermediate values of the components are obtained, suggesting that they follow a mixture rule behavior.

**Figure 1:**
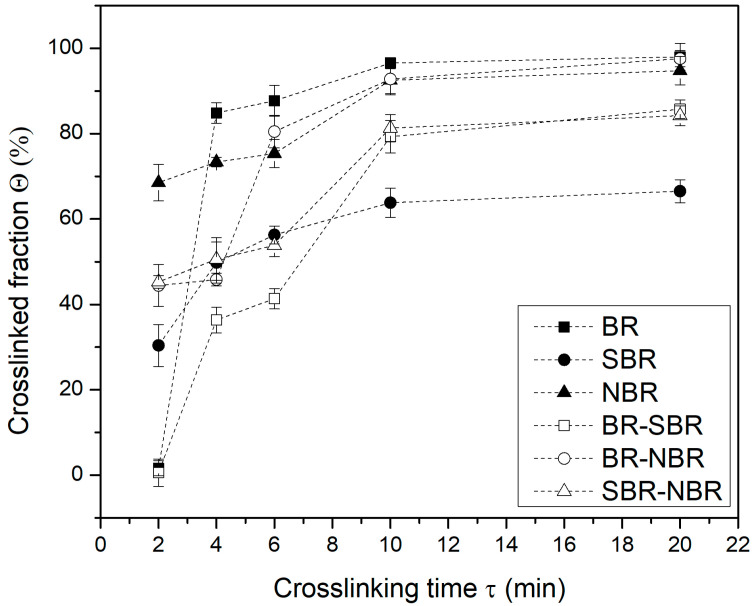
Crosslinked fraction (Θ) versus crosslinking (τ) time for the elastomers and their blends.

Figure 1 shows the crosslinked fraction versus time for the elastomers and their blends. At 2 min, the SBR and NBR samples crosslink rapidly, with values close to 30% and 70%, respectively. At this crosslinking time, the BR presents only about 1% of crosslinking. This behavior may be caused by the difference in the reaction rate of DCP in each of the elastomers, suggesting that this rate is in the following order: NBR > SBR > BR. However, at 4 min of crosslinking, BR is now the elastomer with the highest crosslinked fraction, while SBR shows the lowest percentage of crosslinking. On the other hand, the NBR that initially crosslinked very quickly, after 4 min, has increased its crosslinked fraction by only 5%. At the end of the crosslinking time (20 min), it can be observed that the BR and NBR were almost completely crosslinked (Θ = 1), while the SBR only reached 60% of crosslinked fraction. All this can be explained by the number of active sites or double bonds in the chains of the different elastomers. For example, when observing the chemical structure of each of the elastomers, for BR there is one double bond for every four carbons, while for NBR there is one double bond for every seven carbons, and for SBR, one for every twelve carbons.

**Figure 2:**
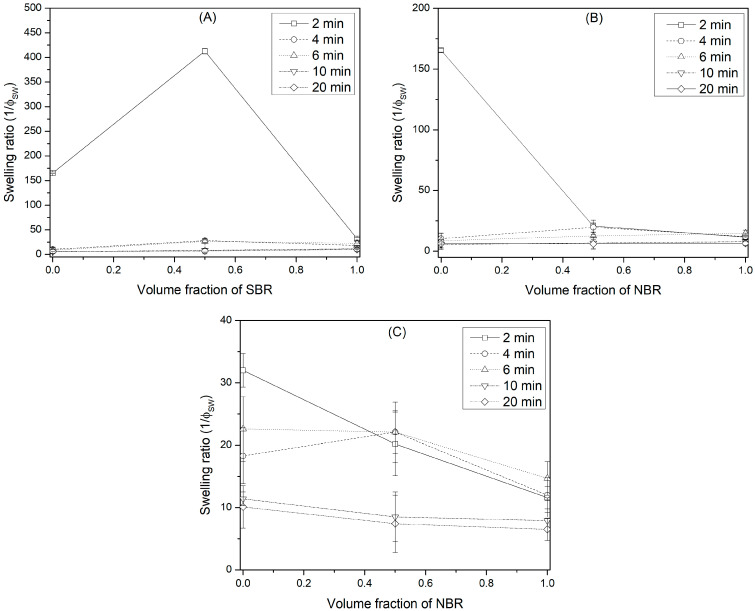
Effect of mixing the elastomers on the swelling ratio for each of the blends: (**A**) BR-SBR, (**B**) BR-NBR and (**C**) SBR-NBR.

Table S1 includes the results of the calculated swelling ratio. In general, all elastomers and their blends presented a similar behavior, where the swelling ratio decreased with crosslinking time. Consequently, the highest swelling values occurred at the shortest crosslinking times. Among the elastomers, BR presented the highest swelling values, while NBR was the least swollen. Figure 2 shows the effect of blending the elastomers on the swelling ratio. The mixing effect showed interesting results, since for some of the blends, a positive deviation from the rule of mixtures was observed. That is, there was an increase in swelling for certain crosslinking times. For example, the BR-SBR blend crosslinked for 2 min presented a swelling value of 412 times, while the individual BR and SBR elastomers presented swelling values of 165 and 32, respectively. This blend presented the same behavior at crosslinking times of 4 and 6 min, but the swelling values were not as high. The same performance was presented by the BR-NBR and SBR-NBR blends at 4 min of crosslinking time. According to Marzzoca et al. [40], while two rubbers may be virtually insoluble, the blend prepared by intense mechanical means can be macroscopically homogeneous, and once the blend has been crosslinked, it can perform as a single-phase system. On the other hand, according to Wang [41], the PS domains within the SBS are more continuous for the sample processed in an internal mixing chamber than for the sample obtained by casting. This was observed by transmission electron microscopy (TEM) as a hexagonal structure for the PS domains in the sample prepared by casting (SBS_I_), while in the samples prepared by compression (SBS_II_) and in the internal mixing chamber (SBS_III_), the microstructure was more distorted, and almost no hexagonal domains of PS were observed. These observations suggested a phase separation in the samples SBS_II_ and SBS_III_. Although phase separation may exist at the microscopic level, the mixture can behave as a single phase at the macroscopic level due to the effect of crosslinking. On the other hand, hemp fibers have been used to mechanically reinforce ethylene–propylene–diene–terpolymer (EPDM), which hindered the absorption of solvent within the blend, decreasing the degree of swelling and increasing the crosslink density [3].

### 4.2. Tensile Tests of the Elastomers and Their Blends

Figure S1 shows the stress–strain curves for the elastomers and their blends. For pure elastomers, as the stress increases, the deformation increases, until reaching a maximum where the specimen breaks. The NBR specimen presented the maximum deformation, being approximately 500%, while the BR presented the minimum with a value of 4%. The mixtures behave in the same way, where the BR-NBR specimen reached up to 300% deformation. On the other hand, it is evident that, at low strains, the stress value is higher for samples that contain a greater number of crosslinks. Thus, samples with longer crosslinking time become more rigid. In this sense, BR specimens were the most rigid, since they presented the lower deformation, while the most elastic were the NBR specimens.

**Figure 3:**
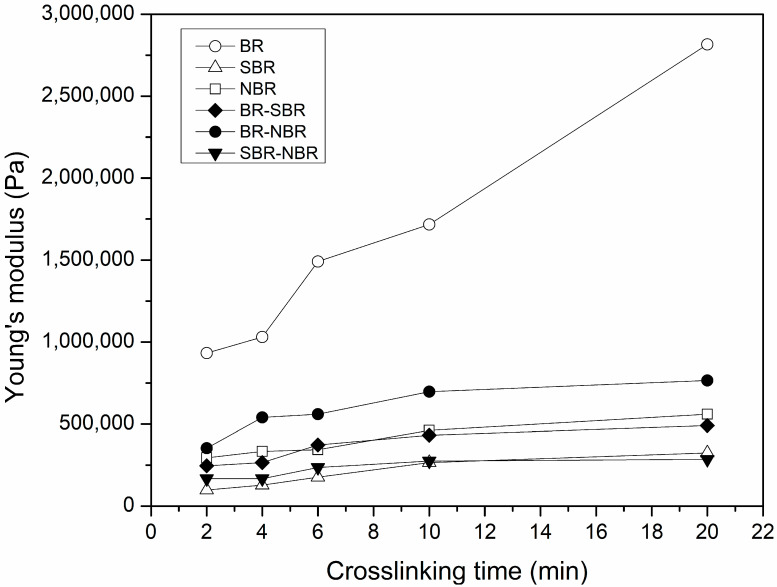
Young’s modulus values versus the crosslinking time for the elastomers and their blends.

Young’s modulus was determined from the linear region of the stress–strain curve for each of the specimens. Young’s modulus values are presented in Table S1. Figure 3 shows Young’s modulus values versus the crosslinking time for the elastomers and their blends. For all samples, by increasing the crosslinking time, the Young’s modulus increases. With the exception of the BR-SBR samples, the other elastomers and blends presented very similar values and slope. However, the BR-SBR sample presented the highest values of Young’s modulus and a more pronounced slope. Evidently, this is caused by the differences in the crosslinking fraction previously described. Similar results were reported by Smejda-Krzewicka et al. for blends of chloroprene (CR) and BR, crosslinked with zinc oxide [31]. They found that the blend with the lowest swelling degree presented both the highest crosslinking density and tensile strength.

For the blends, the effect of adding another elastomer reduces the Young’s modulus, which indicates a negative deviation from the rule of mixtures. This effect is more evident at higher crosslinking times, while it is attenuated as the crosslinking time decreases. This behavior can be explained in terms of the difference in diffusivity of the DCP in each of the elastomers during the mixing and crosslinking stages. Nah et al. found that the velocity of the initiator and an accelerator in NBR is lower than that for natural rubber (NR) [42]. Furthermore, if during mixing, the initiator diffuses into both phases, and during crosslinking, the DCP preferentially migrates to one of the phases, then crosslinking will be favored within one of the phases. Gardiner et al. employed an optical analysis to investigate the diffusion of crosslinking substances in elastomer blends. They found that the migration of these substances is related to diffusion during crosslinking and not to transfer during mixing [43]. The addition of SiO_2_ nanoparticles in mixtures of polypropylene (PP) and polyolefin elastomer (POE) promotes phase refining, which significantly increases the Young’s modulus [44].

### 4.3. Average Molecular Weight between Crosslinks

**Figure 4:**
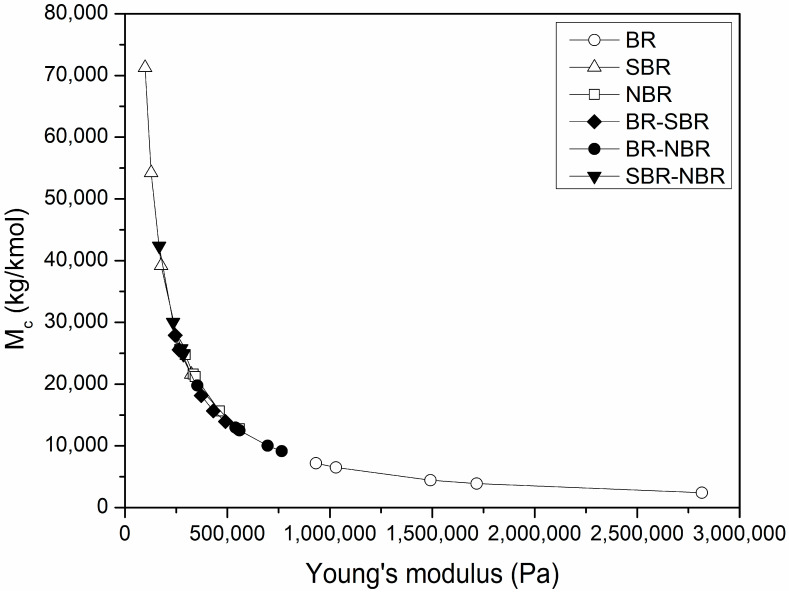
M_c_ values plot against the Young’s modulus for the elastomers and their blends.

It is well known that crosslinked polymers absorb when immersed in organic solvents; the volume and absorption rate will depend on the temperature, molecular weight of the solvent, crosslinking density of the polymer and the polymer–solvent interactions, in addition to the added additives [45]. Hence, the determination of the average molecular weight between crosslinks (M_c_) is important to predict other properties. Then, based on the knowledge of the Young’s modulus and Equation (9), the M_c_ value was calculated for each of the elastomers and their blends. These values are reported in Table S1. Figure 4 shows the M_c_ values plot against the Young’s modulus. An increase in Young’s modulus is observed as the length of the M_c_ chain decreases. The shape of the curve is due to the inverse relationship between M_c_ and Young’s modulus. The decrease in M_c_ indicates the occurrence of more crosslinking events of shorter average length, caused by the increase in the crosslinking time. The decrease in M_c_ results in an increase in Young’s modulus, causing the material to become more rigid. In this sense, BR sample showed the lowest M_c_ values, while the SBR sample showed the highest values. The M_c_ values for NBR and the blends remained in between. In mixtures of NBR with polyvinyl chloride (PVC), an inverse relationship between Mc and Young’s modulus was also found. This behavior was attributed to the formation of weak Van der Waals bonds at a concentration of 40 phr of PVC [46].

### 4.4. Determination of the Flory–Huggins Interaction Parameter

Once the values of M_c_ and 1/ϕ_sw_ have been calculated, it is possible to solve the Flory–Rehner equation for X_12_. In this work, the interaction parameter (χ_12_*) will be defined as the resultant parameter of solving Equations (6), (9) and (11). This dimensionless parameter takes into account the specific interactions between the polymer chains of the blend and the solvent molecules for a given polymer–solvent system [47]. Likewise, the Flory–Huggins criterion for complete miscibility of the polymer and the solvent throughout the composition range is χ_12_ < 0.5.

**Figure 5:**
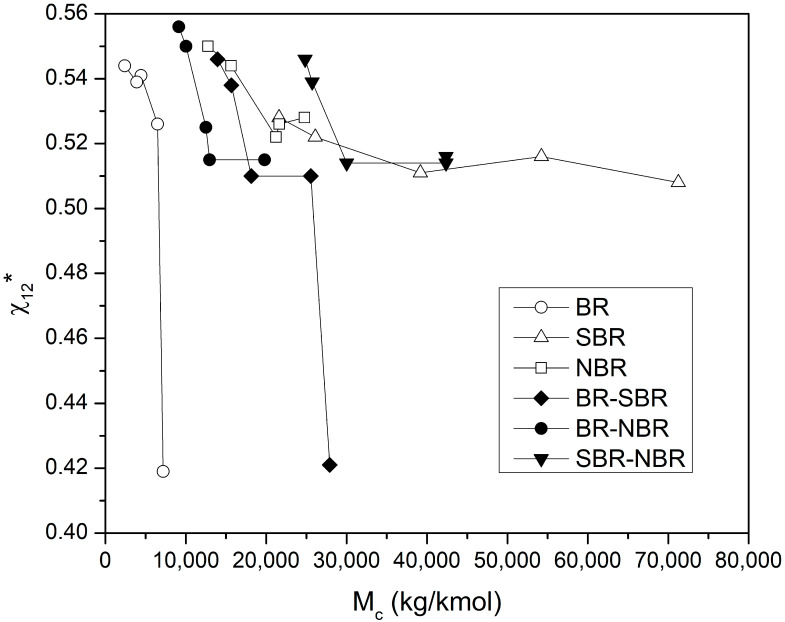
Interaction parameter (χ_12_*) against the chain length M_c_, for the elastomers and their blends.

Figure 5 shows the interaction parameter (χ_12_*) against the chain length M_c_, for the elastomers and their blends. Overall, χ_12_* tends to decrease as M_c_ increases, which is presented at short crosslinking times, or low crosslinking fractions. Most of the χ_12_* values are slightly higher than the miscibility criterion, indicating the low interaction between the solvent and the elastomers and blends. The only two points below the miscibility criterion correspond to the BR and BR-SBR samples, both with a crosslinking time of 2 min. The spread of the points on the graph suggests an inverse dependence between χ_12_* and M_c_, indicating that for crosslinked elastomers and their blends, there is a narrow range of interaction parameters, rather than a single value. Table 2 shows the range of χ_12_* values obtained at different values of crosslinking fractions. Likewise, for comparative purposes, values of χ calculated from the solubility parameters expressed in Equation (5) are included. All the values calculated herein are slightly higher than those calculated from the solubility parameters. Equation (5) only allows to introduce a single polymer for the calculation of the interaction parameter, and consequently, the resulting value is valid only for the polymer–solvent system employed. Additionally, strictly speaking, whenever specific interactions such as those with hydrogen bonds and donor–acceptor interactions are involved, the solubility parameter concept must fail [35]. Therefore, the values of χ calculated from Equation (5) and reported in Table 2 should be considered as reference values for comparative purposes only and not as absolute values. On the other hand, the Flory–Rehner equation provides the change in free energy upon swelling of a polymer gel. The derived interaction parameter (χ_12_*) is a quantitative measure of the degree of interaction between the crosslinked polymer and solvent molecules [48,49]. A lower value implies higher polymer–solvent interaction and thus indicates higher solubility.

Several works have also reported different values of the interaction parameter for the same material. Martter et al. evaluated the interaction parameter for a series of branched and star-type polybutadienes, concluding that the interaction parameter (i) does not vary monotonically with the number of arms in the star, (ii) decreases with increasing concentration of the star and (iii) is almost invariable with increasing the length of the linear PB chain [50]. Schwahn et al. presented the calculation of the interaction parameter from experiments in a low-angle neutron diffractometer (SANS), for a series of mixtures of polybutadiene with polybutadiene of different vinyl content and different molar volume. Among their results, they reported that the interaction parameter has a strong inverse dependence on the molar volume [51]. Alberda et at., through DSC studies, experimentally determined the phase separation of blends of polystyrene with poly(styrene-*co*-4-vinylpyridine) and poly(styrene-*co*-2-vinylpyridine), and calculated the interval of the interaction parameter as 0.30 < χ_S-4VP_ < 0.35 and 0.09 < χ_S-2VP_ < 0.11, respectively [52]. Okay et al. prepared GDP with sulfur monochloride at low crosslinking concentrations, and reported a three-term equation to estimate the interaction parameter as a function of the volume fraction of the polymer [53]. Carbognani et al. studied asphaltenes swollen with polar and non-polar hydrocarbons, from which they obtained the solubility parameters of asphaltenes [54]. They proposed the use of an iterative method to simultaneously determine M_c_, χ_12_ and δ_2_, which is based on the assumption that each asphaltene has a single pair of M_c_ and δ_2_ values. The χ_12_ values obtained in this way were lower than those obtained experimentally and from the literature. Habeeb Rahiman and Unnikrishnan studied the SBR-NBR system at different compositions and with three different crosslinking agents (sulfur, DCP and mixed). They determined that in the presence of chlorinated hydrocarbons, the value of χ is always higher for the blends crosslinked with DCP, and lower when sulfur was used. Likewise, the value of χ can vary, depending on the polarity of the solvent and the SBR content in the blend. The reported that values of χ were found in the range of 0.6–0.64 and 0.36–0.42, for carbon tetrachloride (CCl_4_) and chloroform (CHCl_3_), respectively [22]. Paul and Ebra-Lima reported the interaction parameter for crosslinked pure gum rubber membranes in the presence of 12 organic solvents. Among these solvents, the reported values of χ for benzene and CCl_4_ were 0.474 and 0.353, respectively [55].

### 4.5. Evaluation of the Thermodynamic Parameters

**Figure 6:**
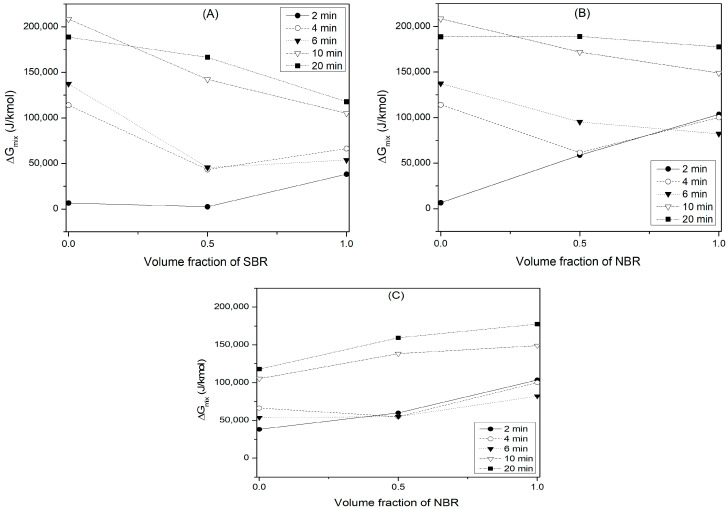
Effect of blending on the ΔG_mix_ of the elastomer blends. (**A**) BR-SBR, (**B**) BR-NBR and (**C**) SBR-NBR.

After the interaction parameter for the elastomers and their blends has been estimated, it is possible to determine the values of the thermodynamic parameters (ΔG_mix_, ΔH_mix_, and ΔS_mix_) from Equations (1)–(3) or by solving the Flory–Huggins relationship from Equation (4). Table S2 shows the calculated values of the thermodynamic parameters, while Figure 6 shows the ΔG_mix_ for the elastomer blends. The ΔG_mix_ values are positive, which means that the swelling phenomenon does not take place spontaneously. However, ΔG_mix_ tends to decrease at low crosslinking times, suggesting more spontaneous swelling. Positive ΔG_mix_ values indicate moderate swelling, where 0 < 1/ϕ_sw_ < 1. For blends, the ΔG_mix_ is lower than for individual elastomers. This effect is more prominent at low crossing times (2, 4 and 6 min), while at times greater than 10 min, the behavior is the opposite. For example, the blends of BR-SBR at 2, 4 and 6 min of crosslinking, BR-NBR at 4 min, and SBR-NBR at 4 and 6 min presented this behavior.

The enthalpy values are positive and tend to increase with crosslinking time, indicating that the solvent absorption phenomenon is an endothermic process. In an endothermic process, the formation of a site for the subsequent absorption of the solvent is necessary first [56]. Thus, the swelling can be improved by increasing the temperature of the system.

The ΔS_mix_ is always positive and tends to increase with crosslinking time. However, the values are relatively low, suggesting few configuration modes of the polymer chains between the two elastomers in the blend. On the other hand, when comparing the values of ΔS_mix_ against those of ΔH_mix_, a low contribution of ΔS_mix_ on the ΔG_mix_ was observed which results in positive values of this thermodynamic parameter.

## 5. Conclusions

The thermodynamic parameters (ΔG_mix_, ΔH_mix_ and ΔS_mix_) of crosslinked elastomers of immiscible nature and their blends were reported herein. The study was based on a phenomenological approach to determine the Flory–Huggins interaction parameter (χ_12_*), from swelling experiments and tensile tests. The samples prepared at shorter crosslinking times presented a greater swelling ratio and lower values in the Young’s modulus. The effect of mixing on the swelling ratio showed a positive deviation from the rule of mixtures for crosslinking times lower than 6 min. The calculated values of the average molecular weight between crosslinks (M_c_) decrease with the Young’s modulus, indicating that the longer the crosslinking time, there is a greater occurrence of crosslink bonds but of shorter average length. The χ_12_* showed values slightly above the miscibility criterion (χ_12_ < 0.5), an inverse relationship with M_c_, as well as a range of values instead of a single value for the same elastomer–elastomer solvent system. The determination of the ΔG_mix_ indicated that the swelling phenomenon of elastomers and their blends is not a spontaneous process, while the ΔH_mix_ showed the endothermic nature of the absorption process. The mixing of the elastomers showed a positive effect at low values of crosslinking time, where the blends presented lower ΔG_mix_ values than those of the individual components.

## Figures and Tables

**Table 1:** Sample name and content of the blends.

Sample	BR (g)	SBR (g)	NBR (g)	DCP (g)
P	60	0	0	0.6
S	0	60	0	0.6
N	0	0	60	0.6
PS	30	30	0	0.6
PN	30	0	30	0.6
SN	0	30	30	0.6

**Table 2:** Range of values of χ_12_* at different crosslinking fractions for the elastomers and their blends.

Sample	Range of χ_12_*	Crosslinking Fraction (Θ)	χ Calculated from Equation (5)
BR	0.447–0.569	0.01–0.98	0.4369
SBR	0.510–0.535	0.3–0.66	0.4039
NBR	0.524–0.557	0.68–0.94	0.3449
BR-SBR	0.434–0.553	0.006–0.85	0.4204
BR-NBR	0.516–0.561	0.44–0.97	0.3909
SBR-NBR	0.515–0.550	0.45–0.84	0.3744

## Data Availability

Some of the results and the discussion reported herein are portions of the master’s thesis in materials science for C.L.-P. presented in 2004, which can be consulted in the institutional repository at the link: “https://cimav.repositorioinstitucional.mx/jspui/”.

## Supplementary Materials

The following supporting information can be downloaded at: https://www.mdpi.com/article/10.3390/polym16030351/s1, Figure S1: Stress–strain curves for the elastomers (BR, SBR and NBR) and their blends (BR-SBR, BR-NBR and SBR-NBR); Table S1: Sample identification and crosslinking time (τ). Values determined experimentally: Weights from the Soxhlet extraction process, crosslinking density (Θ), swelling ratio (Φ_sw_), density (ρ) and Young’s modulus (YM). Calculated values for parameters M_c_ and χ_12_*; Table S2: Calculated values of the thermodynamic parameters.
