# Supplementary material for: Thermodynamic Parameters of Crosslinked Elastomers (BR, SBR and NBR) and Their Blends
Source: Polymers (Basel). 2024 Jan 28;16(3):351. doi: 10.3390/polym16030351 (PMC10856988; doi:10.3390/polym16030351)
Supplement: Supplementary file 1 [file polymers-16-00351-s001.zip › polymers-2757807-supplementary.pdf]

polymers

MDPI

Supplementary Materials

Thermodynamic Parameters of Crosslinked Elastomers (BR, SBR and NBR) and Their Blends

César Leyva-Porras *, Iván A. Estrada-Moreno *, Claudia I. Piñón-Balderrama, Sergio G. Flores-Gallardo and Alfredo Márquez-Lucero

Advanced Materials Research Center (CIMAV), Complejo Industrial Chihuahua, Miguel de Cervantes No. 120, Chihuahua 31136, Mexico; claudia.pinon@cimav.edu.mx (C.I.P.-B.); sergio.flores@cimav.edu.mx (S.G.F.-G.); alfredo.marquez@cimav.edu.mx (A.M.-L.)

* Correspondence: cesar.leyva@cimav.edu.mx (C.L.-P.); ivan.estrada@cimav.edu.mx (I.A.E.-M.); Tel.: +52-61-4439-1100 (ext. 2011) (C.L.-P.); +52-61-4439-1100 (ext. 2404) (I.A.E.-M.)

Table S1. Sample identification and crosslinking time (τ). Values determined experimentally: Weights from the Soxhlet extraction process, crosslinking density (Θ), swelling ratio (Φ_{sw}), density (ϱ) and Young's modulus (YM). Calculated values for parameters M_c and χ_{12}*.

Sample	τ (min)	W_1 (g)	W_2 (g)	W_3 (g)	W_4 (g)	Θ (%)	$1/\Phi_{sw}$	ϱ (kg/m^3)	YM (Pa)	M_c (Kg/Kmol)	χ_{12}*
BR	2	1.7305	1.1403	4.71	1.7489	1.61	165.5	900	931950	7181.7	0.419
BR	4	1.9872	1.1046	10	2.9247	84.87	10.4	900	1030050	6497.7	0.526
BR	6	1.8738	1.1367	12.13	2.8709	87.72	8.6	890	1491120	4438.7	0.541
BR	10	1.9529	1.0811	8.22	2.9967	96.55	5.5	900	1716750	3898.6	0.539
BR	20	1.8247	1.1774	8.11	2.9786	98.00	6.1	910	2815470	2403.6	0.544
SBR	2	2.0266	1.1198	12.26	2.367	30.40	32	940	98100	71259.1	0.508
SBR	4	1.8264	1.1519	14.16	2.4001	49.80	18.3	930	127530	54231.5	0.516
SBR	6	2.0193	1.1282	13.05	2.6543	56.28	22.6	930	176580	39167.2	0.511
SBR	10	1.9437	1.1135	9.67	2.6544	63.83	11.4	930	264870	26111.5	0.522
SBR	20	1.9145	1.1451	9.2	2.6762	66.52	10.1	940	323730	21593.6	0.528
NBR	2	1.8842	1.107	9.87	2.6428	68.53	11.6	980	294300	24763.8	0.528
NBR	4	1.7058	1.1414	12.86	2.5432	73.37	12	970	333540	21627.4	0.526
NBR	6	1.7403	1.0816	10.69	2.5554	75.36	14.7	980	343350	21226.1	0.522
NBR	10	2.042	1.095	9.44	3.0553	92.54	7.9	970	461070	15645.3	0.544
NBR	20	1.979	1.1274	8.53	3.0473	94.76	6.5	960	559170	12767.5	0.550
BR-SBR	2	2.2939	1.136	4.98	2.3007	0.60	412.9	920	245250	27897.1	0.421
BR-SBR	4	1.5169	1.1572	12.99	1.9374	36.34	28.1	910	264870	25549.9	0.510
BR-SBR	6	1.83	1.0899	13.52	2.2806	41.34	26.7	910	372780	18153.9	0.510
BR-SBR	10	1.7616	1.1397	9.08	2.6654	79.30	8.3	910	431640	15678.3	0.538
BR-SBR	20	1.8749	1.0335	7.9	2.7609	85.73	7	920	490500	13948.5	0.546
BR-NBR	2	1.79	1.1579	11.79	2.3043	44.42	20.7	940	353160	19794.2	0.515
BR-NBR	4	1.9493	1.1241	11.49	2.4644	45.82	19.7	940	539550	12956.2	0.515
BR-NBR	6	1.9988	1.032	11.89	2.8294	80.48	12.6	940	559170	12501.6	0.525
BR-NBR	10	2.134	1.1225	8.86	3.1758	92.81	6.8	940	696510	10036.4	0.550
BR-NBR	20	2.2045	1.0866	8.39	3.2647	97.57	6.1	940	765180	9135.7	0.556
SBR-NBR	2	1.7688	1.1227	11.36	2.2772	45.28	20.2	950	166770	42363	0.516
SBR-NBR	4	1.7356	1.1467	13.7	2.316	50.61	22.1	950	166770	42363	0.514

SBR-NBR	6	1.6807	1.145	14.36	2.2971	53.83	22.1	950	235440	30007.1	0.514
SBR-NBR	10	2.0103	1.082	9.08	2.8902	81.32	8.5	950	274680	25720.4	0.539
SBR-NBR	20	1.8111	1.0842	8.14	2.7243	84.23	7.4	950	284490	24833.5	0.546

W_1 is the initial weight of the extraction capsule, W_2 is the weight of the elastomer sample, W_3 is the weight of the swollen elastomer in the extraction capsule, and W_4 is the dried weight of the remaining elastomer in the capsule after the extraction process.

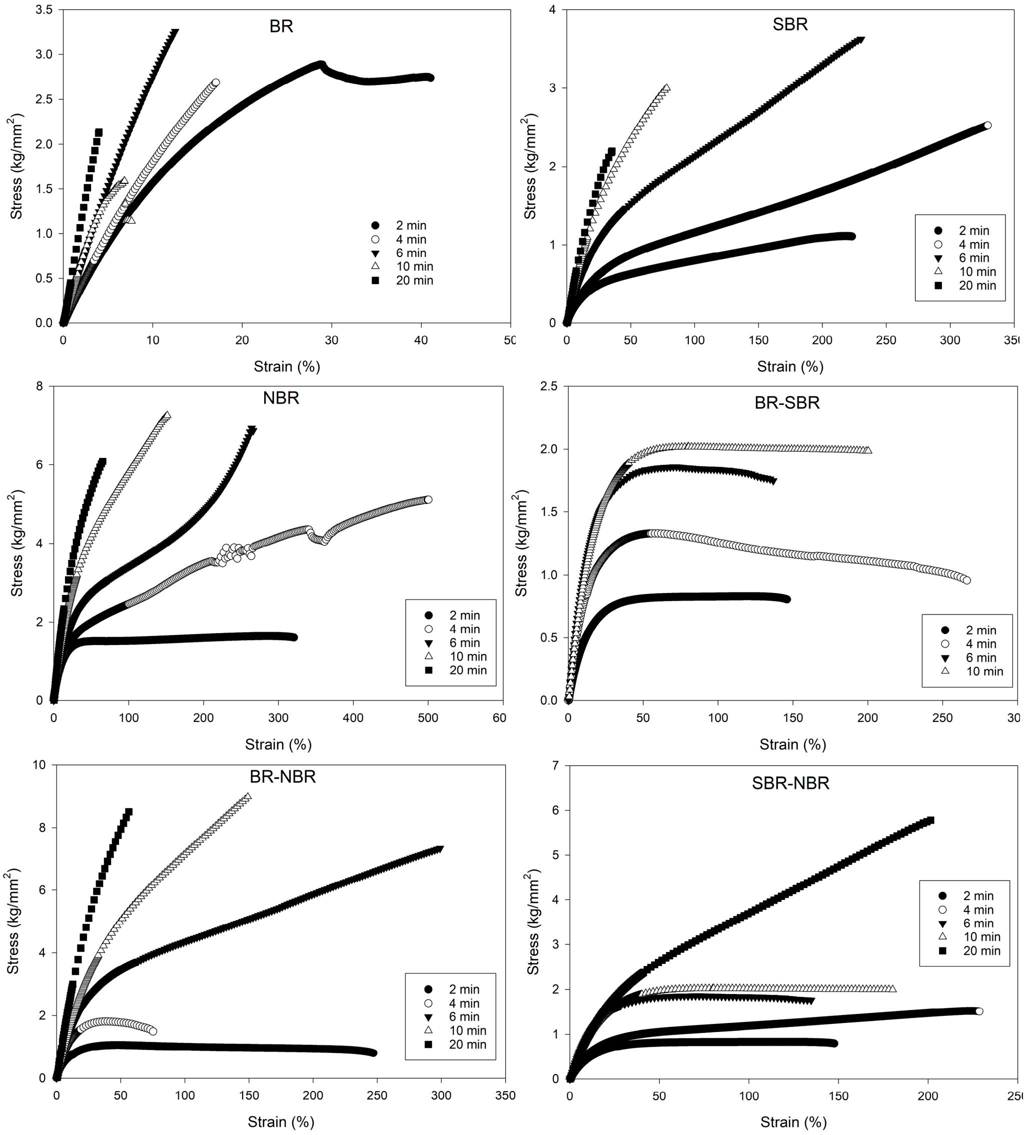

Figure S1. Stress-strain curves for the elastomers (BR, SBR, and NBR) and their blends (BR-SBR, BR-NBR, and SBR-NBR).

Tables S2. Calculated values of the thermodynamic parameters.

Sample	τ (min)	ΔG_{mix} (J/Kmol)	ΔS_{mix} (J/Kmol)	ΔH_{mix} (J/Kmol)
BR	2	6645.12	0.15	6690.53
BR	4	114005.23	40.31	126023.85
BR	6	137521.82	60.51	155561.63
BR	10	208487.03	153.86	254358.95
BR	20	188680.70	122.46	225191.52

SBR	2	38242.43	4.13	39475.21
SBR	4	66431.83	12.88	70272.97
SBR	6	53828.41	8.34	56315.08
SBR	10	105073.92	33.79	115149.51
SBR	20	117866.88	43.23	130754.84
NBR	2	103515.62	32.74	113276.33
NBR	4	100241.25	30.58	109358.06
NBR	6	82171.15	20.10	88164.51
NBR	10	148959.89	71.96	170414.63
NBR	20	177501.79	106.37	209215.11
BR-SBR	2	2596.80	0.02	2604.08
BR-SBR	4	43383.81	5.36	44982.54
BR-SBR	6	45578.96	5.94	47349.74
BR-SBR	10	142256.29	65.03	161645.64
BR-SBR	20	166493.74	92.12	193957.90
BR-NBR	2	58810.63	10.02	61799.57
BR-NBR	4	61663.11	11.07	64963.64
BR-NBR	6	95192.83	27.42	103369.58
BR-NBR	10	171883.50	98.96	201387.03
BR-NBR	20	189165.21	122.91	225810.80
SBR-NBR	2	60033.34	10.45	63147.87
SBR-NBR	4	54999.55	8.72	57598.80
SBR-NBR	6	55101.10	8.76	57711.75
SBR-NBR	10	138245.22	61.07	156453.33
SBR-NBR	20	159320.93	83.48	184210.81
